# Challenges and Limitations of Endocrine Toxicity Evaluation in Non-Small Cell Lung Cancer Patients Treated with Immunotherapy—Retrospective Study from a Tertiary-Level Hospital in Romania

**DOI:** 10.3390/diagnostics13101788

**Published:** 2023-05-18

**Authors:** Simona Coniac, Mariana Cristina Costache Outas, Edvina-Elena Pirvu, Raluca-Ileana Patru, Estera Gainariu, Ciprian Aldea, Polixenia Georgeta Iorga, Mihaela Ambroci, Horia-Dan Liscu, Andreea-Iuliana Miron, Corin Badiu

**Affiliations:** 1 Department of Medical Oncology, Coltea Clinical Hospital, 030167 Bucharest, Romania; simona.horlescu@drd.umfcd.ro (S.C.);; 2 Department of Endocrinology, Faculty of Medicine, “Carol Davila” University of Medicine and Pharmacy, 020021 Bucharest, Romania; 3 Department of Endocrinology, Coltea Clinical Hospital, 030167 Bucharest, Romania; 4 Department of Medical Oncology, Bucharest Emergency University Hospital, 050098 Bucharest, Romania; 5 Department of Medical Oncology, Hôpital Paul-Brousse, 94804 Villejuif, France; 6 Department of Radiotherapy, Coltea Clinical Hospital, 030167 Bucharest, Romania; 7 Discipline of Oncological Radiotherapy and Medical Imaging, “Carol Davila” University of Medicine and Pharmacy, 020021 Bucharest, Romania; 8 Department of Endocrinology, “C.I. Parhon” National Institute of Endocrinology, 011863 Bucharest, Romania

**Keywords:** endocrine immune-related adverse events (irAEs), immune checkpoint inhibitors (ICIs), Romania

## Abstract

(1) Background: The endocrine system has become a prominent target to autoimmune damage during treatment with immune checkpoint inhibitors (ICIs) in cancer patients. Real-world data regarding endocrine immune-related adverse events (irAEs) are needed to explore their impact in cancer patients. An analysis was conducted to evaluate endocrine irAEs caused by ICIs, besides the challenges and limitations of daily medical practice in oncology in Romania. (2) Methods: This was a retrospective cohort study of lung cancer patients treated with ICIs at Coltea Clinical Hospital, Bucharest, Romania, from 1 November 2017 to 30 November 2022. Endocrine irAEs were identified through endocrinological assessment and were distinguished as any occurring endocrinopathy during treatment with ICIs and related to immunotherapy. Descriptive analyses were performed. (3) Results: Of 310 cancer patients treated with ICIs, we identified 151 with lung cancer. From this cohort, 109 NSCLC patients qualified for baseline endocrine estimation and 13 patients (11.9%) developed endocrine irAEs, such as hypophysitis (4.5%), thyroid disorder (5.5%) and primary adrenal insufficiency (1.8%), with one or more endocrine glands being affected. There might be a correlation between endocrine irAEs and duration of ICI treatment. (4) Conclusions: Early diagnosis and adequate management of endocrine irAEs may be challenging in lung cancer patients. A high incidence of endocrine irAEs is expected with the growing use of ICIs, and because not all endocrine events are immune-related, cooperation between oncologists and endocrinologists is crucial in the management of these patients. More data are needed to confirm the correlation between endocrine irAEs and the efficacy of ICIs.

## 1. Introduction

Endocrine immune-related adverse events (irAEs) have emerged as an individual group of rather frequent and highly anticipated toxicities of immunotherapy. Diverse studies report different incidences, but elevated incidence is probably due to superior recognition of the condition and more experience with immunotherapy [1]. Immune checkpoint inhibitors (ICIs) weaken the cytotoxic T-lymphocyte-associated antigen 4 (CTLA-4) and the programmed cell death 1 (PD-1)/ligand 1 (PD-L1) pathways, and modulate the ligand–receptor interactions between cancer cells and the patient’s immune cells within the tumor microenvironment, depriving cancer cells of a key strategy of evasion from immunosurveillance. It is more often reported that patients treated with anti-CTLA4 antibodies have an increased risk of developing hyphophysitis and patients treated with anti-PD-1/PD-L1 antibodies illustrate a higher risk of primary thyroid dysfunction [2].

Regardless of the thoroughly described mechanisms of action of immune checkpoint inhibitors (ICIs) [3], the pathogenesis of endocrine irAEs remains elusive. In contrast to other immune-mediated adverse events, endocrine irAEs have a tendency to convert to irreversibility and to result in the need for lifelong hormonal replacement therapy [4]. Why irreversible? An appealing proposed explanation would be that endocrine fragility comes from increased vulnerability to an autoimmune attack, as endocrine glands are small tissue reservoirs disseminated all over the body and are less likely to regenerate and restore function [5]. Furthermore, there is an escalating curiosity to better identify patients prone to developing endocrine irAEs. Research in the literature discloses future perspectives of predictive and sensitive biomarkers that could be useful in clinical studies, such as thyroid autoantibodies and cytokines/chemokines [6]. Algorithms of screening, monitoring, diagnosis and management of endocrine toxicities during ICI treatment have also been proposed in the last few years by the European Society of Medical Oncology [7,8,9].

The cumulative awareness of endocrine irAEs is justified by the significant statistical correlation between endocrine toxicities and progression-free survival (PFS) during ICIs. Recent meta-analysis and retrospective studies on immunotherapy in lung cancer patients validated the predictive power of endocrine irAEs of enhanced ICI efficacy and overall survival [10,11,12].

In our retrospective study, we aimed to identify the challenges and limitations of endocrine toxicity evaluation in routine clinical practice in lung cancer patients treated with ICIs in a tertiary-level hospital in Romania. The predictive impact of endocrine irAEs should be explored in a non interventional, observational phase IV study by a multidisciplinary team.

## 2. Materials and Methods

We performed a retrospective cohort study, approved by the ethical institutional review board, of adult locally advanced or metastatic non-small cell lung cancer (NSCLC) patients treated with immune checkpoint inhibitors (ICI) at Coltea Clinical Hospital, Bucharest, Romania, for a period of 5 years from 1 November 2017 (first PD-1 inhibitor used at our institution, Nivolumab) until 30 November 2022. We assessed the baseline characteristics of NSCLC patients, type of immunotherapy treatment and type of response using registered data. For endocrine toxicity evaluation, we integrated exclusion criteria to avoid bias, such as patients with a history of cervical radiotherapy due to head and neck cancer or lymph node metastasis, patients with just one ICI administration and those lost in follow-up. Patients must have been evaluated for thyroid functional tests (TFTs) at baseline and a minimum of one follow-up value. We did not intend to evaluate diabetes mellitus as endocrine immune-related adverse event. Oncologist specialist requested endocrinology evaluation based on modified TFTs or high clinical suspicion of endocrine dysfunction, such as sudden onset of headache of inexplicable cause, digestive intolerance (nausea, vomiting and diarrhea), inexplicable weight loss, orthostatic hypotension with vertigo, hyponatremia.

Endocrine irAEs were characterized as any occurring autoimmune endocrinopathy during treatment with ICIs and related to immunotherapy. Endocrine adverse events not related to immunotherapy were evaluated separately. Patients’ evaluation, diagnostics and treatment were performed by the endocrinologist specialist from our institution. Thyroid laboratory testing was performed at the Coltea Clinical Hospital Laboratory, Bucharest, Romania and included screening for autoimmune thyroiditis i.e., TSH, fT4, T3, and ATPO. Adrenal function reserve was evaluated using basal cortisol and ACTH as well as low dose (1 ug) ACTH stimulation test with cortisol measurements at 30 and 60 min. Our laboratory’s reference ranges for adults for thyroid-stimulating hormone (TSH) are 0.465–4.68 mIU/L (VITROS_ECI), 0.55–4.78 mIU/L (ATTELICA) and 0.465–4.68 mIU/L (VITROS 7600), respectively, and for free thyroxine (FT4) are 10–28.2 pmol/L (VITROS_ECI), 11.5–22.7 pmol/L (ATTELICA) and 10–28.2 pmol/L (VITROS 7600). Cortisol laboratory reference ranges are 123–626 nmol/L (blood sampling must be performed before 10:00 a.m.) and 46.2–389 nmol/L (blood sampling must be performed after 5:00 p.m.) (VITROS 7600). Results of other endocrine tests, if needed and especially requested by endocrinologist, such as thyroid peroxidase (TPO) antibodies, adrenocorticotropic hormone (ACTH), follicle stimulating hormone (FSH), luteinizing hormone (LH) and prolactin, were assessed in external clinic and interpreted based on validated reference ranges.

The main outcomes of the study were descriptive analyses of real-world NSCLC patients treated with ICIs according to NIH protocols in Romania, medical reasons of oncologist to request endocrinology assessment, patients’ access to comprehensive endocrine evaluation, and natural history and frequency of endocrine irAEs in locally advanced or metastatic NSCLC patients treated with ICIs. The secondary endpoint was to take into consideration the probability of any correlation between endocrine irAEs and time of ICI treatment.

## 3. Results

Of 310 cancer patients treated with ICIs at Coltea Clinical Hospital, Bucharest, Romania, over a period of 5 years, we identified 1 case of breast cancer, 3 of cancer of unknown primary, 12 of renal cancer, 10 of urothelial cancer, 42 of malignant melanoma, 91 of head and neck cancer and 151 of lung cancer, respectively, treated with PD-1 inhibitor (nivolumab—142 patients, pembrolizumab—146 patients), PD-L1 inhibitor (atezolizumab—10 patients, durvalumab—2 patients, avelumab—1 patient) and CTLA-4 antibody (ipilimumab—9 patients). For our retrospective analysis, being the largest population in the database, we selected lung cancer patients for a homogeneous group. We did not combine cancer populations in order to avoid bias from different types of treatments, such as cervical radiotherapy in head and neck cancer patients, or different natural histories and overall survival, such as malignant melanoma patients.

### 3.1. Study Population

#### 3.1.1. Lung Cancer Population at Baseline

Adult lung cancer patients treated with immunotherapy for a period of 5 years in our institution comprised 147 NSCLC patients and 4 small cell lung cancer (SCLC) patients, which were subsequently excluded from this analysis. This cohort counted 104 males and 43 female adults, with a median age of 65 years old. Smoking status and occupational exposure to carcinogens were not always registered in medical documents; thus, we calculated 63 former smokers (42.8%) and 18 patients with professional exposure to toxics. Only 10 patients (6.8%) were aware of hereditary cancers (6 patients with family history of lung cancer). Histology features discovered 99 patients with adenocarcinoma and 48 patients with squamous cell carcinoma. All the patients (145) were in a metastatic stage before ICI treatment. Only two patients were treated with durvalumab as consolidative immunotherapy for unresectable stage III NSCLC. A number of 39.4% of patients had at least one metastatic site before ICI treatment, and 28.5% had more than three sites. Pulmonary metastasis was most frequently identified (53.7%), followed by bone sites (32.6%). Brain metastases were present in 28 patients (19.0%). Adrenal metastasis was described in radiological results in 22 patients (14.9). Demographic and disease characteristics of NSCLC patients at the baseline, before starting immunotherapy, are thoroughly presented in Table 1.

PD-L1 expression was acknowledged from medical data as TPS (tumor proportion score, meaning a report between PD-L1 positive tumor cells and the number of PD-L1 positive and PD-L1 negative tumor cells). The PD-L1 test was positive in 71 patients (48.2%) and not assessed in 28 patients (19%). Two-thirds of patients were treated with pembrolizumab (62.5%).

#### 3.1.2. Lung Cancer Population after Treatment with Immune Checkpoint Inhibitor

NSCLC patients treated with ICIs comprised 145 patients, as patients treated with Durvalumab were excluded (different ICI therapeutic usage indication). Only seven patients were oncogene-driven and treated as such, according to ESMO guidelines (in force at actual time of treatment), before immunotherapy. Ten patients received radiotherapy for previous head and neck or cancer of unknown primary or synchronous head and neck cancer. More NSCLC patients (63.5%) were treated with immunotherapy in first line indication. Disease and treatment characteristics of the cohort population treated with ICI are detailed in Table 2.

We did not divide patients treated with first-line monotherapy versus chemotherapy plus immunotherapy because the purpose of this study was to evaluate all patients treated with ICI for immune-related adverse events that can occur any time during treatment, or even long after stopping ICI. There were 21 patients (14.4%) treated with just one administration of ICI and lost in the follow-up.

ICI proved efficacy for more than half of the patients (53.7%), and the average time on immunotherapy was 7.8 months. Response to ICI treatment was estimated from imaging results (most always CT scans) and conveyed to fulfill National Insurance House protocol requirements. We used terminology such as “complete response”, “partial response”, “stable disease”, “clinical benefit” and “progressive disease” without being able to provide RECIST (response evaluation criteria in solid tumors) measurements. Nevertheless, only 21 patients (14.4) presented with progressive disease, and 31% were not assessed for response to ICI.

#### 3.1.3. Lung Cancer Population Analyzed for Endocrine Immune-Related Adverse Events

**Figure 1:**
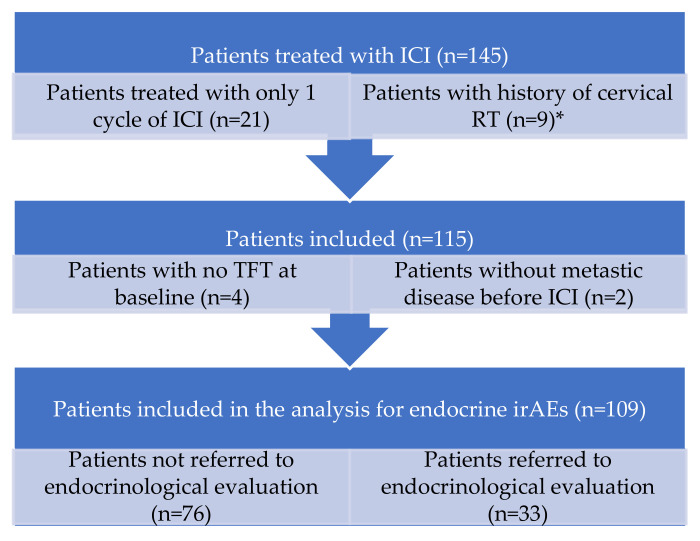
Overview of the retrospective cohort of ICI-treated metastatic NSCLC patients to identify the occurrence of endocrine immune related events. * One patient had been treated with cervical RT and also just one cycle of treatment.

To meticulously appraise endocrine immune-related adverse events, we expanded the evaluation of the initial cohort of 145 NSCLC patients. Therefore, we excluded patients treated with just one cycle of immunotherapy (21 patients; of these, 11 patients did not have TFT assessed as the baseline), patients without TFT at the baseline (another 4 patients) and patients with a history of cervical radiotherapy (10 patients, but 1 patient also had just one ICI cycle). Additionally, for homogeneous reasons, two NSCLC patients without metastatic disease were excluded. Nevertheless, it is worth mentioning the proportion of patients treated with pembrolizumab—68 patients (62.3%); nivolumab—38 patients (34.8%); and Atezolizumab—3 patients (2.75%). This consecutive-step decision making is represented in Figure 1.

Disease and treatment characteristics of this final population of metastatic NSCLC patients evaluated for irAEs (such as demographics, histological features, metastasis sites, PD-L1 expression, response to ICI treatment and prior and subsequent treatments) are thoroughly described in Table 3. Two-thirds of the patients had histologic features of adenocarcinoma. Brain metastasis was present in 22 patients and bone secondary sites were present in 35.7% of patients. PD-L1 was positive in 45.8% of patients. The median time of immunotherapy was estimated as 8.9 months. ICI efficacy was revealed in 61.4% of the patients, as detailed in Table 3. Radiotherapy was performed for tumor and mediastinal lymph nodes in 41 patients, for brain metastasis in 28 patients and for bone metastasis in 18 patients, during the entire course of treatment, before or after immunotherapy. Patients could benefit from several methods of radiotherapy according to curative or palliative purposes, and to the maximum allowed dosage. Patients were treated with platinum-doublet chemotherapy (CHT), especially in first-line metastatic disease and in monotherapy in a second-line setting. Chemotherapy was recommended as a standard guideline at the physician’s choice, and included docetaxel, gemcitabine, navelbine, paclitaxel and pemetrexed.

### 3.2. Analysis of Endocrine Adverse Events

The dynamics of thyroid functional tests during the study is thoroughly shown in Table 4. As described, the majority of NSCLC patients treated with ICIs had normal baseline TFTs, but during treatment, these TFTs started to modify, and 20.1% of patients encountered high or low TSH values. Nine patients with modified TFT during treatment normalized spontaneously. Almost 70% of patients in this specified analysis did not need endocrinologist evaluation.

The summary of clear-cut criteria for irAEs and not-related endocrine adverse events is presented in Table 5. 

Instead, 30% (meaning 33 patients) were assessed by the endocrine specialist in our institution for two reasons: monitoring, or suspicion of endocrine immune-related adverse events. Three patients in the monitoring group (six patients totally) either presented with pre-existing endocrine dysfunctions (one patient with autoimmune thyroiditis; one patient in etiological investigation for central hypothyroidism, hyperprolactinemia and secondary adrenal insufficiency after steroid treatment; and one patient with micropolynodular thyroid) or were examined as a baseline evaluation before ICI treatment (three patients).

Table 6 summarizes the main endocrine events testified in our study.

In the final analysis, 14 patients were diagnosed with endocrine adverse events not related to immunotherapy. Thyroid dysfunction occurred in three patients due to iodine contrast agent (two cases of hyperthyroidism and one hypothyroidism case); one patient presented hyperprolactinemia due to rib bone metastasis injury; one case of hypercalcemia happened in one patient due to bone metastasis; two cases of primary adrenal insufficiency were developed after steroid treatment. One case of thyroid dysfunction (hypothyroidism) was prone to lymph node metastasis. Two patients had more than one gland concurrently affected. Three patients did not encounter any endocrine dysfunction. Interestingly, only four patients needed hormone replacement treatment.

**Figure 2:**
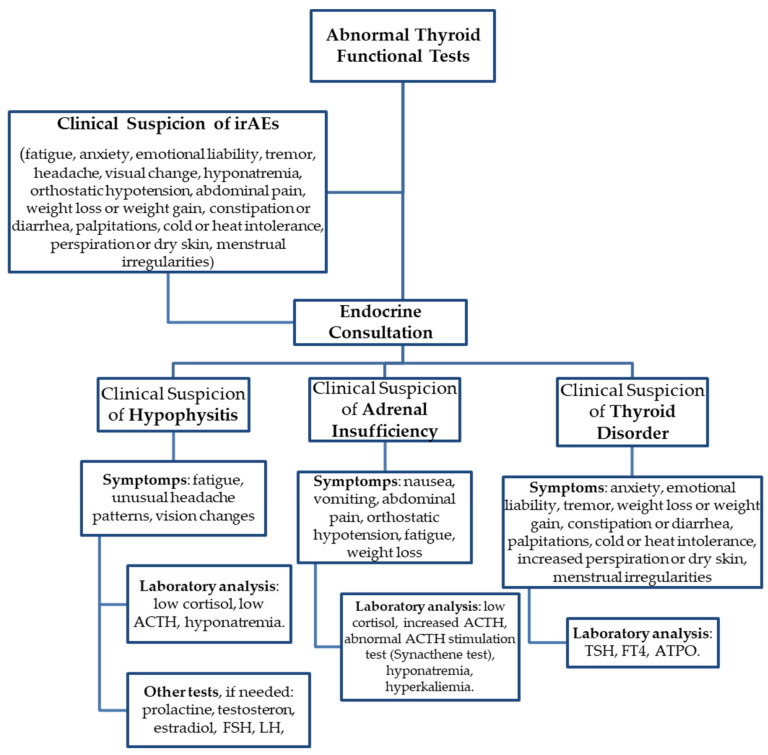
Overview of algorithm of irAE diagnostic process.

Endocrine immune-related adverse events were reported in 13 patients (11.9) in this study—8 patients treated with Pembrolizumab and 5 patients with Nivolumab, respectively—and comprised 5 cases of hypophysitis (4.5%), 5 patients with hypothyroidism (4.5%) and 1 patient with hyperthyroidism. Primary adrenal insufficiency (PAI) was diagnosed in two patients. The median time till the onset of endocrine irAEs was 4.6 months (range 0–10 months). The average time on immunotherapy for NSCLC patients evaluated for endocrine irAEs was 8.9 months, which is 1.1 months longer than in the overall population of the study (7.8 months, as described above). According to the Common Terminology Criteria for Adverse Events (CTCAE) [13], all endocrine irAEs were classified as Grade 1 or 2, without indication to permanently stop immunotherapy. Eleven patients (84.6%) imposed hormonal replacement therapy, indefinitely. One or more glands could be affected. The course of endocrine immune-related adverse events, from suspicion to diagnosis and management, are specified in Figure 2 and Table 7, Table 8, Table 9 and Table 10. None were life-threatening. The Pearson correlation coefficient between endocrine irAEs and duration of ICI treatment described a *p* value < 0.001, which is strongly statistically significant.

### 3.3. Statistical Analysis

We performed a simple binary logistic regression in order to identify possible predictors associated with irAEs, and the predictors that were statistically significant were introduced in a multiple binary logistic regression model to determine the overall effect of this predictors. To investigate the factors that can influence patient outcome, we performed a simple Cox regression, and for factors with significant influence, we performed a survival analysis in order to calculate RMST (“restricted mean survival time”) and median survival time. We also tested the survival curves using a log-rank test. All predictors that were statistically significant in the simple Cox regression were used in a multiple Cox regression to calculate the overall effect of these predictors. The significance level α for this study was 0.05, so *p*-values less than 0.05 were considered statistically significant. For statistical analysis, the R program, version 4.2.3, was used (Copyright (C) 2023 The R Foundation for Statistical Computing, R Core Team (2023)). (R: A language and environment for statistical computing. R Foundation for Statistical Computing, Vienna, Austria. URL https://www.R-project.org (accessed on 7 May 2023).

Compared to patients with elevated levels of TSH during treatment, patients with normal and decreased levels of TSH had a 30-fold reduction in odds of developing irAEs. The period of ICI administration was associated with an increase in odds of developing irAES; a one-week increase was associated with a two-percent increase in odds of developing irAEs. The amount of series of ICIs was also associated with an increase in odds of developing irAES, with a four-percent increase in odds of developing irAEs for a single supplementary series of ICIs, as shown in Table 11.

In the multiple-predictor model, a single supplementary series was associated with a 14% increase in odds of developing irAEs, but a one-week increase in ICI duration was associated with a 7% decrease in odds of developing irAEs, as presented in Table 12.

**Figure 3:**
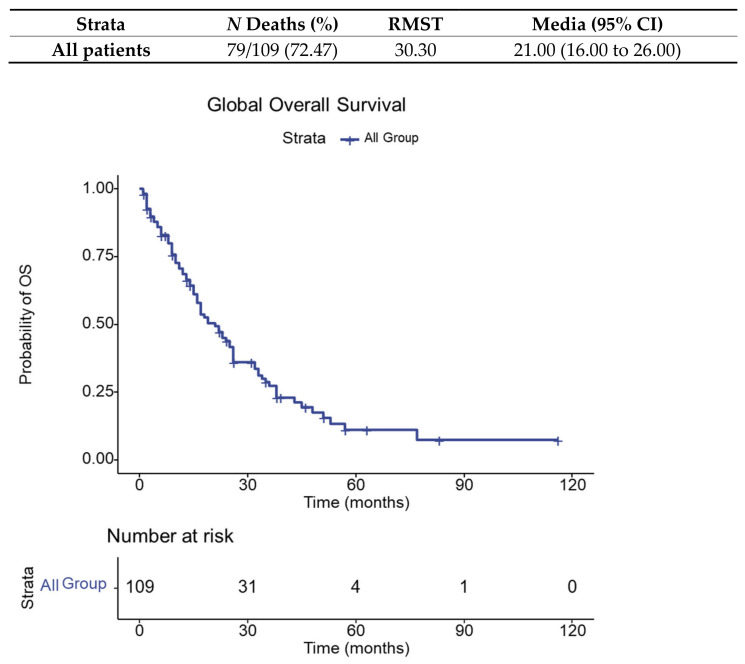
Global overall survival.

Survival analysis is expressed by overall survival (RMST = restricted mean survival time) and illustrated below for the entire sample of metastatic NSCLC patients evaluated for irAEs (109 patients), in Figure 3.

**Figure 4:**
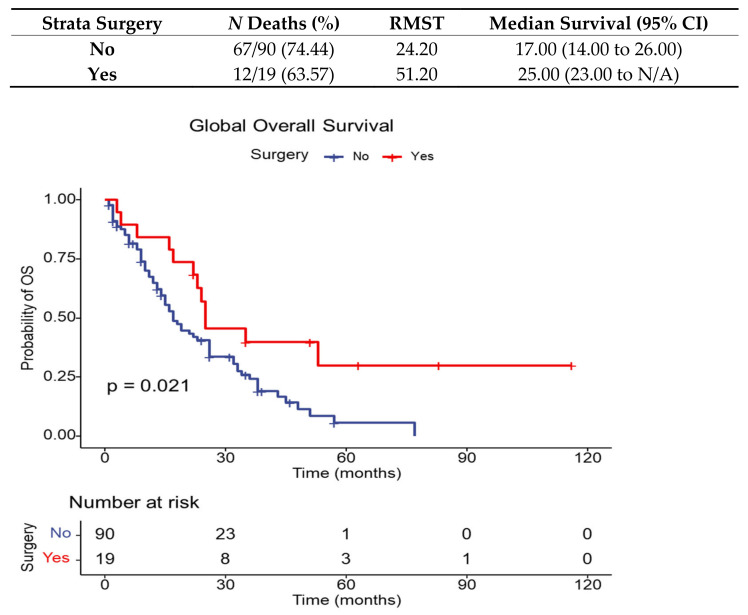
Overall survival for all NSCLC patients and for patients treated with surgery before ICI.

We identified three factors that can influence the outcome (death) of metastatic NSCLC patients: an increased age (a one-year increase was associated with a three-percent increase in hazard ratio); curative surgery, which was associated with a fifty-percent reduction in hazard ratio; and occurrence of irAEs, associated with a seventy-four-percent reduction in hazard ratio. Predictors of the outcome (death) are presented in Table 13. The overall survival for patients treated with surgery in their medical history for NSCLC disease is revealed below in Figure 4.

**Figure 5:**
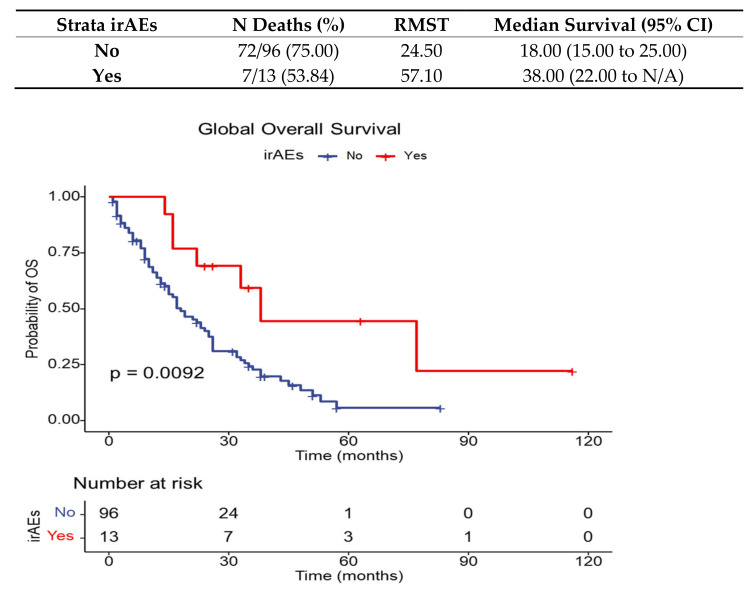
Overall survival for metastatic NSCLC patients with irAEs compared to global population in the study.

The overall survival for metastatic NSCLC patients evaluated in our retrospective study for irAEs is presented in Figure 5.

The predictors we identified at the multiple Cox regression were curative surgery and irAE development, which are both associated with a decrease in hazard ratio. These predictors of the outcome (death) are revealed in Table 14.

## 4. Discussion

The tremendous effective success of immunotherapy in many malignancies over the last decade has become a routine medical practice in oncology. Nevertheless, discrepancies in different countries may occur. Starting with one major predicament, in Romania, the first ICI reimbursed by the National Insurance House (NIH) and used in NSCLC patients was nivolumab [14] in November 2017 [15], and only approved indication was as second-line monotherapy; this is the only used indication of Nivolumab till today. During the next five years, other ICIs entered into clinical practice, such as pembrolizumab in September 2018 [16], just in first-line monotherapy indication [17]; atezolizumab in July 2021 [18] in second-line monotherapy indication [19]; and finally, durvalumab [20], in February 2022 [21]. This reality explains the small proportion of NSCLC patients treated with atezolizumab and durvalumab in our institution in the selected period of this retrospective analysis. Cemiplimab is not reimbursed by NIH for use in NSCLC patients in Romania [22]. Nowadays, to simplify the process of searching for the reimbursed indication of ICIs throughout the abundant approved European Medicines Agency (EMA) market authorizations, one should check the Romanian Medical Oncology Society’s site [23] and follow legal steps to correctly fill in an NIH file. Limitations in treating NSCLC patients with all the extensive and effective new antibodies narrow the horizon of patient’s clinical benefits. First-line nivolumab plus ipilimumab combined with two cycles of chemotherapy indication in NSCLC patients [24,25] was finally revised and actually during process of approval for reimbursement by NIH since April 2023. Nivolumab was not available in this indication in our study.

Secondly, but equally as important for therapeutic decision making, molecular tests should be carried out; however, in Romania, they are not reimbursed by NIH. European Society of Medical Oncology (ESMO) guidelines recommend molecular tests in advanced non-squamous cell carcinoma and in unusual cases of squamous cell carcinoma [26]. Standard molecular tests include EGFR mutation status and ALK rearrangements (level of evidence I, A). Testing should also include ROS1 rearrangements, BRAF V600 mutation status, NTRK rearrangements, MET exon 14 skipping mutations, MET amplifications, RET rearrangements, KRAS G12C mutations and HER2 mutations (level of evidence II, A). PD-L1 expression should be systematically determined in advanced NSCLC, and PD-L1 testing is required for first-line pembrolizumab and atezolizumab monotherapy, as well as second-line pembrolizumab (level of evidence I, A) [27]. In Romania, provided in the form of sponsored vouchers by pharmaceutical companies, minimal biomarker testing comprises EGFR mutation, ALK rearrangements and PD-L1 expression, according to NIH-supported medicines. Limited access to diagnosis and comprehensive biomarker panels in Romania were recently reported by The Swedish Institute for Health Economics, which analyzed access to cancer drugs in Europe [28].

Furthermore, NSCLC patients treated with ICIs should be evaluated for response. Measurements following response evaluation criteria in solid tumors (RECIST) v1.1 should be used [29]. In real-world clinical practice, RECIST evaluation is not always detailed in imaging reports (CT scans in the majority of cases). Consequently, the oncologist must judge the response to ICI based on radiologist conclusions, especially to properly assess maintenance criteria for continuing ICI treatment in NIH records. The difficulty for cancer patients to access imaging centers, low-income affordability and time-consuming schedules reduce the opportunity for consistent radiological evaluation. Thus, sporadic evaluation may happen, and may elucidate the proportion of NSCLC patients from our retrospective study who were not assessed for ICI response, which was 30%.

In our retrospective study, we decided to perform several exclusion criteria for the NSCLC cohort to be appraised for endocrine dysfunctions. The rationale behind these constrictive exclusion criteria was to better identify patients to evaluate for endocrine toxicities and avoid bias. Thyroid dysfunction assessment consists of baseline and dynamic TFT, so patients without baseline TFT were excluded. Cervical radiotherapy for head and neck cancers is well known as a bias in thyroid function abnormalities [30,31,32], so it was a pertinent omission judgement. In daily clinical practice, referral to endocrinology evaluation is elective. As endocrine irAEs have been reported in randomized clinical trials [33,34,35,36,37,38] and in updated systematic reviews and meta-analyses [39,40], ESMO guidelines recommend TFT at the baseline every 4–6 weeks during treatment, as well as 4–6 weeks after the last cycle [9].

Endocrine toxicities were detected in all ICI regimens in a large FDA adverse-event-reporting system [41]. We do not have experience in our retrospective study with anti-CTLA4/PD-L1 antibodies or combinations, so all endocrine irAEs reported in this analysis are related to anti-PD-1 antibody treatment.

Thyroid dysfunction is one of the most common endocrine irAEs in NSCLC patients treated with anti-PD-1 antibodies [42]. Moreover, in our study, thyroid toxicity was the most frequently reported endocrine irAE (6 out of 13 patients). Brilli et al. testified a significant association between the development of overt thyroid dysfunction and both TSH and positive antithyroid antibody (ATAb) levels at the baseline [43]. In our study, 97% of patients had normal baseline TSH levels, and ATAb were not performed, as it is not a routine clinical practice. On the other hand, early hypothyroidism during ICI could be predicted by higher baseline TSH levels [44]. We reported five patients with hypothyroidism, and all of them presented normal TSH baseline values and needed lifelong replacement therapy. Another study, which reported a high incidence of thyroid disorders (21%) with a PD-L1 inhibitor, concluded that elevated thyroid peroxidase (TPO) antibodies at the time of thyroid irAEs might impact the gravity of thyroid dysfunction, thus helping to identify patients who will progress to overt hypothyroidism and require thyroid hormone replacement [45]. We did not assess TPO antibodies at the baseline as it is not possible in routine oncological practice. The same study outlined that diffusely increased thyroids on ^18^-fluorodeoxyglucose–positron emission tomography–computed tomography (^18^FDG-PET/CT) may predict the occurrence of thyroid dysfunction. This observation is supported by another review that acknowledged the ability of ^18^FDG-PET/CT to detect autoimmune thyroiditis [46]. Although the normal thyroid gland usually does not express high 18F-FDG uptake, there is an increased incidental finding of thyroiditis detected by PET/CT. The mechanism of underlying uptake in autoimmune thyroiditis is not well understood, but could possibly be clarified by the activated lymphocyte microenvironment in the infiltrated thyroid. The author also concluded that the detection of thyroiditis by ^18^FDG-PET/CT may become a prognostic marker. Supplementarily, the probability of immune-mediated thyroiditis associated with a better response to immunotherapy is even stronger if radiologic manifestations are present (evaluated through Doppler ultrasound or conventional thyroid scintigraphy with technetium Tc-99 m pertechnetate imaging methods) [47]. Wrapping up, clinical research suggests that thyroiditis may be a biomarker for antitumor immune response, emphasizing the need to further characterize its underlying mechanism. In Romania, there are distinctive eligibility conditions for reimbursed ^18^FDG-PET/CT in the National Oncology Programme [48], which was prohibitive to our study setting.

Finally, higher TSH levels and the presence of antithyroglobulin autoantibodies (TgAbs) and/or antithyroid peroxidase autoantibodies (TPOAbs) might be used as pre-treatment biomarkers and TgAbs and/or TPOAbs increase and thyroglobulin (Tg) elevation might be applied as during-treatment predictive biomarkers [49]. The clinical practice value of these specified biomarkers might hypothetically predict thyroid irAEs and manage them appropriately. As mentioned above, in daily clinical practice, we did not assess TgAbs or TPOAbs as pretreatment biomarkers, and we assessed them as during-treatment biomarkers only if indicated by the endocrinologist.

Primary adrenal insufficiency (PAI) is a rare endocrine irAE [50], and adrenal crisis can be life-threatening, so increased awareness of this endocrine toxicity should be a main apprehension in oncological routine clinical practice for patients treated with ICIs. Nonspecific symptoms such as fatigue, nausea, hypotension, anorexia and dyselectrolytemia in laboratory testing (i.e., hyponatremia, hyperkalemia and hypoglycemia) are usually common in cancer patients in a metastatic setting. So, regular measurements of serum cortisol and adrenocorticotropin (ACTH) could be recommended for cancer patients treated with immunotherapy to smooth an early diagnosis [51]. In our institution, ACTH laboratory testing is not routinely rated, so it was inaccessible as part of the baseline, and exclusively evaluated on the endocrinologist’s request during the irAE diagnostic algorithm. If necessary, the ACTH test was provided externally. Given the special conditions of transport and storage for ACTH evaluation, no samples were collected in our institution. Another imperative argument in interpreting adrenal disorder in metastatic NSCLC patients is the high tumor burden in adrenal metastasis. In our retrospective study, we reported 22 patients with adrenal metastatic sites and 1 patient with PAI related to this. In randomized clinical trials [33,34,35,36,37,38], there was no description of adrenal metastasis. Subsequently, in real-life medical practice, it is vital to properly identify the etiology of PAI. Even though steroids are formally not indicated during ICI treatment (with concessions for a small dose of prednisone) in everyday oncological practice, urgent situations to use steroids are common. In our report, we define two cases of PAI due to steroid treatment. In this perspective, we emphasize the magnitude of ruling out steroid treatment and adrenal metastasis in cancer patients treated with ICI for accurately recognizing immune-related PAI.

A meta-analysis from 2018 reported hypophysitis more often as endocrine irAE related to anti-CTLA-4 antibodies and a low incidence of 0.4% of patients treated with anti-PD-1 antibodies [52]. As mentioned before, we do not report experience with anti-CTLA-4 antibodies in this retrospective analysis. In the selected NSCLC population revised for endocrine irAEs, we identified five cases of hypophysitis (4.5%) that could be explained by the superior doubt of a more experienced endocrinologist in our institution. A recent overview of pituitary disorder proposed insightful recommendations for magnetic resonance imaging (MRI) to confirm hypophysitis and to disregard differential diagnoses, especially pituitary metastasis [53]. Although moderate pituitary enlargement can be rapidly reversible, ICI-induced hypophysitis should not be ruled out due simply to normal imaging. For patients treated with anti-PD-1/anti-PD-L1 agents, no abnormalities on MRI may be common, and this was one potential reason why hypophisitis in patients treated with PD-1/PD-L1 agents might have been underestimated [41]. In our study, we did not report MRI imaging for hypophisitis, as this imaging method is not a routine-based clinical practice. A retrospective study from Greece that included thyroid and pituitary gland irAEs proved statistical significance of progression-free survival and overall survival for cancer patients treated with immunotherapy, concluding that endocrine toxicity may be a positive predictor of ICI response [54]. In that context, predictive biomarkers of pituitary gland irAEs are of great interest in the medical community [55], but are not always of clinical utility, and no biomarker has been proven to effectively foresee the likelihood of evolving a specific endocrine irAE after ICI [49]. The pathophysiological pattern and underlying mechanism of anti-PD-1/anti-PD-L1 antibody-related hypophysitis were projected, and incriminated type IV hypersensitivity as the main pathway [56]. Currently, there is an innovative focus of medical research and an emerging concept of onco-immuno-endocrinology. Immune-related hypophysitis is described as paraneoplastic autoimmune hypophysitis, a novel clinical entity [57].

Immunotherapy in metastatic NSCLC patients substantially improved the prognosis and survival benefit of these patients. The existing reported correlation between immune-related adverse events and improved response to immune checkpoint inhibitors was corroborated with higher survival rates [58]. In conjunction with the fact that most irAEs can be successfully managed, it became crucially important to identify the patients at risk of adverse events in a timely manner. Predictive and sensitive biomarkers might be useful to adequately stratify the risks in these patients and to monitor them closely for early detection and treatment. Several cytokines, and chemokines such as IL-2, IL-8, granulocyte colony-stimulating factor (G-CSF), IFN-γ, TNF-α, granulocyte–macrophage colony-stimulating factor (GM-CSF), monocyte chemoattractant protein (MCP-1), antithyroglobulin autoantibody (TgAb) and anti-TPO autoantibody (TPOAb), have been identified to be significantly interrelated with thyroid irAEs [6]. In a recently informed review, Shalit et al. defined pretreatment and during treatment biomarkers for each endocrine dysfunction, such as specific human leukocyte antigen (HLA) alleles, antipituitary antibodies (APAs) and anti-GNAL abs (anti-guanine-nucleotide-binding protein G(olf) subunit alpha antibodies) for pituitary dysfunction [49]. Remarkably, higher levels of absolute eosinophilic count might have predictive values for endocrine irAEs. We illustrated the biomarkers that we used as baselines and for monitoring, such as TSH and FT4, which are common knowledge in routine clinical practice. Regrettably, we did not have data to publish for TPOAb, ACTH, FSH and LH values to all the patients and not in a monitoring setting by all means. Clinical judgment and vast experience in the oncological background of the specialist endocrinologist in our institution led the management of these patients.

All endocrine irAEs that occurred and were described in our retrospective study were guideline-based treated and follow-up supervised [59,60]. In a real-life setting, it is essential to perfectly diagnose endocrine adverse events, as not all of them are immune-mediated, as our study entirely revealed.

In contrast with all the data presented above, we must stress that an ESMO open systematic review and meta-analysis of randomized trials found no statistically significant correlations between the ICI therapy effects on specific irAEs (i.e., endocrine) [61]. Further data is needed to integrate these results.

The main limitations of our study stem from its retrospective nature, which cannot exclude potential confounders and is also not as accurate regarding the estimation of PFS and other parameters as prospective clinical trials. The retrospective design narrowed the opportunity of extended research and was restrained just to medical information filled up in the archive. It is a well known potential selection bias in a retrospective study but we used this method for database evaluation. As some patients were lost in the follow-up, essential data might have been missed (such as imaging data, laboratory tests, quality of life during ICI). We used several pertinent exclusion criteria to select a homogenous population of NSCLC patients, but this selection could also add potential selection bias. It was a relatively small sample size population evaluated for irAEs, and we did not provide (as they were not available) MRI or imaging data for irAE diagnostics or laboratory analysis of ACTH, ATPO or other biomarkers, as recommended in guidelines. It should also be noted that our study only enrolled patients with NSCLC from Romania, which limits generalizability of the results to other cancer types and/or other countries with potentially different patterns of clinical practice. This single tertiary-level hospital retrospective study might not be in line with medical experience from larger oncological Institutes. In-depth statistical analysis should be provided from this retrospective study to progress the understanding of the prognostic and predictive values of endocrine irAEs in immunotherapy-treated NSCLC patients in Romania.

## 5. Conclusions

Despite the overwhelming data about the likelihood of endocrine irAES predicting immunotherapy efficacy in NSCLC patients, in a real-world setting, careful attention to correctly identify endocrine adverse events related—or not—to ICIs should be a priority. Having experienced endocrinologists in a high-volume cancer patient institution is an indispensable strength to capitalize on the efficacy of ICIs and to balance irAEs. More real-life research is needed to publicly reveal the existing challenges and limitations in different countries and to better identify patients who are at risk of factual immune-related endocrine toxicities. The prognostic and predictive influence of endocrine irAEs in cancer patients should be investigated further in prospective real-life circumstances in a multidisciplinary medical team.

## Figures and Tables

**Table 1:** Demographic and Disease Characteristics of the Patients at Baseline *.

Characteristic	NSCLC Patients Treated with ICIs (*N* = 147)
Age	
Median (range)—years	65 (34–84)
<65 years	68 (46.2)
Male sex—no. (%)	104 (70.7)
ECOG performance status	
0–2	110 (74.8)
3	37 (25.1)
Histologic features—no. (%)	
Adenocarcinoma	99 (67.3)
Scquamous cell carcinoma	48 (32.6)
Number of metastatic sites before ICI—no. (%)	
no site	4 (2.7)
1 site	57 (38.7)
2 sites	44 (30)
3 sites	28 (19.0)
≥4 sites	14 (9.5)
Metastases sites before ICI—no. (%)	
Adrenal	22 (14.9)
Brain	28 (19.0)
Lymph nodes	35 (23.8)
Pulmonary	79 (53.7)
Pleural	42 (28.5)
Liver	30 (20.4)
Bone	48 (32.6)
Other (spleen, skin)	6 (4)
PD-L1 Tumor Proportion Score—no. (%)	
<1%	48 (32.6)
1–49%	30 (20.4)
>50%	41 (27.8)
Not assessed	28 (19)
ICI used—no. (%)	
Atezolizumab	3 (2.0)
Durvalumab	2 (1.36)
Nivolumab	50 (34.0)
Pembrolizumab	92 (62.5)

* Small Cell Lung Cancer Patients were excluded from this analysis.

**Table 2:** Disease and Treatment Characteristics of the Patients after ICI *.

Characteristic	NSCLC Patients after ICIs (*N* = 145) °
Family history of cancer—no. (%)	10 (6.9)
EGFR-mutated NSCLC—no. (%)	7 (4.8)
ALK rearrangements—no. (%)	1 (0.7)
Cervical Radiotherapy—no. (%)	
Primary Head & Neck cancer	4 (2.75)
Synchronously Head & Neck cancer	4 (2.75)
Cancer of Unknown primary	2 (1.37)
ICI Line of treatment—no. (%)	
First line	92 (63.5)
Second line	53 (36.5)
Number of ICI cycles	
Only 1 cycle	21 (14.4)
≥2 ≤6 cycles	55 (37.9)
≥7 ≤12 cycles	25 (17.2)
≥13 ≤19 cycles	15 (10.3)
≥20 cycles	29 (20)
Average time on ICI (range)—months	7.8 (0.5–48.5)
Treatment Response ^1^—no. (%)	
Complete Response	1 (0.7)
Partial Response	29 (20)
Stable Disease	17 (11.7)
Clinical Benefit	1 (0.7)
Partial Response + Stable Disease	25 (17.2)
Partial Response + Stable Disease + Clinical Benefit	1 (0.7)
Partial Response + Clinical Benefit	2 (1.37)
Stable Disease + Clinical Benefit	2 (1.37)
Progressive Disease	21 (14.4)
Not Assessed	46 (31.7)

* Small Cell Lung Cancer Patients were excluded from this analysis. ° Patients treated with Durvalumab in maintenance regimen were excluded from this analysis. Only locally-advanced and metastatic NSCLC patients were included. ^1^ These are criteria for immunotherapy continuation as NIH protocols specify.

**Table 3:** Disease and Treatment Characteristics of the Metastatic NSCLC Patients evaluated for irAEs.

Characteristic	NSCLC Patients Treated with ICIs (*N* = 109)
Age	
>65 years	58 (53.2)
<65 years	51 (46.7)
Male sex—no. (%)	76 (69.7)
ECOG performance status	
0–2	85 (78)
3	24 (22)
Histologic features—no. (%)	
Adenocarcinoma	76 (69.7)
Squamous cell carcinoma	33 (30.2)
Number of metastatic sites before ICI—no. (%)	
1 site	42 (38.5)
2 sites	34 (31.1)
3 sites	23 (21.1)
≥4 sites	10 (9.1)
Metastases sites before ICI—no. (%)	
Adrenal	16 (14.6)
Brain	22 (20.1)
Lymph nodes	27 (24.7)
Pulmonary	59 (54.1)
Pleural	33 (30.2)
Liver	21 (19.2)
Bone	39 (35.7)
Other (spleen, skin)	4 (3.6)
EGFR-mutated NSCLC—no. (%)	5 (4.5)
ALK rearrangements—no. (%)	1 (0.9)
PD-L1 Tumor Proportion Score—no. (%)	
<1%	38 (34.8)
1–49%	21 (19.2)
>50%	29 (26.6)
Not assessed	21 (19.2)
ICI used & line of treatment—no. (%)	
First line—Pembrolizumab	68 (62.3)
Second line	
Nivolumab	38 (34.8)
Atezolizumab	3 (2.75)
Average time on ICI (range)—months	8.9 (0.9–49.9)
ICI Treatment Response ^1^—no. (%)	
Complete Response	1 (0.9)
Partial Response	25 (22.9)
Stable Disease	14 (12.8)
Clinical Benefit	1 (0.9)
Partial Response + Stable Disease	22 (20.1)
Partial Response + Stable Disease + Clinical Benefit	1 (0.9)
Partial Response + Clinical Benefit	2 (1.8)
Stable Disease + Clinical Benefit	1 (0.9)
Progressive Disease	21 (19.2)
Not Assessed	21 (19.2)
Treatment before first line ICI ^1^—no. (%)	
Chemotherapy (platinum doublet)	7 (6.4)
Surgery	19 (17.4)
Radiochemotherapy	2 (1.8)
**First line Pembrolizumab—no. (%)**	**68 (62.3)**
Pembrolizumab Monotherapy	22 (32.3)
Pembrolizumab + Paclitaxel/Platinum doublet ^2^	11 (16.1)
Pembrolizumab + Pemetrexed/Platinum doublet	35 (51.4)
Subsequent treatment after First line Pembrolizumab ^3^	
Pembrolizumab maintenance	35 (51.4)
Monotherapy CHT	16 (23.5)
Platinum doublet CHT	9 (13.2)
**Second line Nivolumab/Atezolizumab—no. (%)**	**41 (37.6)**
First line—Paclitaxel/Platinum doublet ^4^	10 (24.3)
First Line—Pemetrexed/Platinum doublet ^5^	19 (46.3)
First line—Gemcitabine/Platinum doublet	11 (26.8)
First line—CHT monotherapy	1 (2.4)
Subsequent treatment after second line Nivolumab/Atezolizumab ^3^	
Monotherapy CHT	21 (51.2)
Radiotherapy ^6^—no. (%)	
Tumor and mediastinal lymph nodes	41 (37.6)
Brain metastasis	28 (25.6)
Bone metastasis	18 (16.5)
Other metastasis *	1 (0.9)

^1^ Patients were treated with one or more methods. ^2^ One patient received Alectinib before ICI. ^3^ Chemotherapy at physician’s choice, as docetaxel, gemcitabine, navelbine, paclitaxel, pemetrexed. One patient received afatinib. ^4^ One patient received erlotinib and three patients were treated with Paclitaxel/CBDCA/Bevacizumab combination. ^5^ One patient received osimertininb and two patients were treated with afatinib. ^6^ Patients were treated with RT for one or more sites. * Other metastasis treated with RT was retroperitoneal.

**Table 4:** Summary of Thyroid Functional Tests during study.

Characteristic	NSCLC Patients Treated with ICIs (*N* = 109)	
Thyroid Functional Tests *	TSH	FT4
Baseline—no. (%)		
Normal Values	106 (97)	108 (99)
High	1 (0.9)	0
Low	2 (1.8)	1 (0.9)
During ICI—no. (%)		
Normal Values	84 (77)	94 (86)
High	8 (7.3)	2 (1.8)
Low	14 (12.8)	10 (9)
Not assessed	3 (2.7)	3 (2.7)

* 9 patients with modified TFT during ICI presented normal values in follow-up without intervention.

**Table 5:** Summary of clear-cut criteria for irAEs and not-related endocrine adverse events.

Endocrine irAEs	Endocrine Not Related irAEs
Consistent modified TFT	Normalized TFT without treatment
Cortisol levels	Clinical benefit with symptomatic treatment
<137 nmol/L = hypoadrenalism (if not recent documented steroid use)	Accurately assessment of all other etiologies such as:
>400 nmol/L = not PAI	Metastasis sites: M1ADR, M1BRA
ACTH level or Synachten stimulation test	Prior Steroids treatment
High TPOAb	Prior iodinate contrast agent for CT scan
Close endocrinological surveillance	History of cervical or brain radiotherapy

**Table 6:** Summary of Endocrine adverse events during study.

Type of Endocrine Dysfunction	NSCLC Patients Treated with ICIs (*N* = 109)
NO Reason for Endocrine call—no. (%)	76 (69.7)
Reason for Endocrine call—no. (%)	33 (30.2)
Monitoring	6 (5.5)
Suspicion of endocrine events	27 (24.7)
**Endocrine irAEs**	**13 (11.9)**
Hypophysitis	5 (4.5)
Hypothyroidism	5 (4.5)
Hyperthyroidism	1 (0.9)
Primary Adrenal Insufficiency	2 (1.8)
**Not related Endocrine adverse events ^1^** ^,^*^,+^	**14 (12.8)**
Hyperthyroidism	3 (2.7)
Hypothyroidism	3 (2.7)
Hyperprolactinemia	1 (0.9)
Primary Adrenal Insufficiency	3 (2.7)
Hypercalcemia	1 (0.9)
Hypergonadotropic hypogonadism	1 (0.9)
Average time to irAEs—months	4.59 (0–10)

^1^ 1 case of micropolynodular thyroid with suspicion of metastasis in parathyroid gland, clinical euthyroidism was not included in this summary. * Two patients had more than one affected gland. ^+^ Three patients did not encounter any endocrine dysfunction.

**Table 7:** Disease and treatment characteristics of metastatic NSCLC patients that developed irAEs during Immunotherapy.

Nr.	Patient	Sex	Age (Years)	HP	Metastasis before ICI	M1 ADR	M1 BRA	EGFR	ALK	PD-L1	Radiotherapy	ICI	PFS on ICI (Months)	Treatment Response	TSH Baseline	FT4 Baseline	TSH during ICI	FT4 during ICI	TPO Ab	Cortizol	ACTH	Time to irAEs (Months)	Normalized Hormonal Tests with Endocrine Treatment	Endocrine Immune-Related Adverse Events (irAEs)	More Details to irAEs	Endocrine Treatment
1	A.M.	F	66	ADK	2	1	1	NEG	NEG	20%	M1BRA	Nivolumab	2.8	PR	Normal	Normal	HIGH	LOW	NK	LOW	NK	2	YES	Hypothyroidism	Primary Adrenal Insuficiency due to M1Adr	Levothyroxine & Steroids
2	B.V.	B	62	ADK	1	0	0	NEG	NEG	45%	NO	Pembrolizumab	23.1	PR	Normal	Normal	Normal	Normal	NK	LOW	NK	8	YES	Hypophysitis	NO	Steroids
3	B.M.	F	57	ADK	1	0	0	NEG	NEG	5%	NO	Pembrolizumab	13.3	PR	Normal	Normal	LOW	Normal	NK	Normal	NK	6	NA	Subclinical Hyperthyroidism	Functional thyroid tests modified non-specifically. Clinical Euthyroidism	No treatment
4	B.D.	B	55	SC	1	0	0	NEG	NEG	NEG	T/LYM	Pembrolizumab	15.4	PR+SD	Normal	Normal	HIGH	LOW	NK	Normal	HIGH	6	YES	Hypothyroidism	NO	Levothyroxine
5	C.N.	B	66	ADK	4	0	0	NEG	NEG	NK	T/LYM/M1BRA	Nivolumab	6.1	SD	Normal	Normal	HIGH	Normal	NEG	Normal	Normal	6	YES	Subclinical Hypothyroidism	Clinical Euthyroidism	Levothyroxine
6	F.G.	B	57	ADK	2	0	1	NEG	NEG	NK	T/LYM/M1BRA	Nivolumab	10.3	PR+SD	Normal	Normal	Normal	Normal	NK	LOW	NK	9	YES	Primary Adrenal Insufficiency	NO	Steroids
7	M.V.	B	67	ADK	1	0	0	NEG	NEG	15%	M1OSS	Pembrolizumab	10.5	PR	Normal	Normal	Normal	Normal	NK	LOW	NK	10	YES	Primary Adrenal Insufficiency	Clinical Euthyroidism	Steroids
8	N.I.N.	F	51	ADK	2	0	1	NEG	NEG	3%	T/LYM/M1BRA	Pembrolizumab	9.8	PR+SD	Normal	Normal	Normal	LOW	NK	LOW	NK	8	YES	Hypophysitis	Pituitary insufficiency on corticotropic and thyrotropic lines	Levothyroxine & Steroids
9	N.M.	B	52	ADK	2	0	1	NEG	NEG	NK	M1BRA	Nivolumab	45.3	CR	Normal	Normal	Normal	Normal	NK	LOW	NK	5	NA	Hypophysitis	NO	Indication of treatment but lost in follow-up
10	P.V.	B	65	ADK	1	0	1	NEG	NEG	80%	M1BRA	Pembrolizumab	22.4	PR+SD	Normal	Normal	Normal	LOW	NK	LOW	LOW	8	YES	Hypophysitis	Pituitary insufficiency on corticotropic and thyrotropic lines	Levothyroxine & Steroids
11	S.M.	B	78	SC	2	1	0	NEG	NEG	50%	NO	Pembrolizumab	9.8	PR+SD	Normal	Normal	HIGH	Normal	NK	LOW	NK	3	YES	Hypophysitis	Hypothyroidism. Secondary Adrenal Insufficiency due to ICI and M1Adr	Levothyroxine & Steroids
12	S.D.	F	50	SC	4	1	0	NEG	NEG	90%	NO	Pembrolizumab	30.1	PR	Normal	Normal	HIGH	LOW	NK	Normal	NK	2	YES	Hypothyroidism	NO	Levothyroxine
13	T.M.	B	67	ADK	3	0	0	NEG	NEG	NK	T/LYM/M1BRA	Nivolumab	11.7	PR+SD	HIGH	Normal	HIGH	Normal	NK	Normal	Normal	Baseline	YES	Subclinical Hypothyroidism	NO	Levothyroxine

PFS, Progression-free survival; PR, Partial response; SD, Stable disease; CR, Complet response, NA, Not aplicable, NK, Not known, T, Tumour, LYM, Mediastinal lymph nodes, M1Bra, Brain metastasis, M1ADR, Adrenal metastasis, M1OSS, Bone metastasis, ICI, Immune check-point inhibitor. TPO Ab, thyroid peroxidase antibodies; ACTH, adrenocorticotropin; TSH, thyroid stimulating hormone; FT4, free thyroxine.

**Table 8:** Disease and treatment characteristics of metastatic NSCLC patients that developed Endocrine dysfunction not related to Immunotherapy.

Nr.	Patient	Sex	Age (Years)	HP	Metastasis before ICI	M1 ADR	EGFR	ALK	PD-L1	Radiotherapy	ICI	PFS on ICI (Months)	Treatment Response	TSH Baseline	FT4 Baseline	TSH during ICI	FT4 during ICI	TPO Ab	Cortizol	ACTH	Time to Endocrine Consult (Months)	Normalized Hormonal Tests without Endocrine Treatment	Endocrine Dysfunction Not Related to ICI	Endocrine Treatment
1	B.I.	B	64	ADK	1	0	NEG	NEG	NEG	T/LYM	Pembrolizumab	7.0	SD	Normal	Normal	LOW	HIGH	NK	Normal	NK	2	YES	Sublicinical Hyperthyroidism related to iodinated contrast agent	NO
2	B.G.	B	73	ADK	2	0	NEG	NEG	90%	T/LYM	Pembrolizumab	7.7	PR	Normal	Normal	Normal	Normal	NEG	Normal	NK	1	NA	Non-tumor hyperprolactinemia due to rib bone injury and euthyroidism	NO
3	C.I.	B	67	ADK	1	1	NEG	NEG	60%	T/LYM/M1OSS	Nivolumab	2.8	PD	Normal	Normal	LOW	LOW	NK	Normal	Normal	1	NO	Functional thyroid tests modified non-specifically. Minimal subclinical hyperthyroidism. Partial hypocorticism. M1ADR	Steroids
4	C.P.	B	75	ADK	2	1	NEG	NEG	NK	NO	Nivolumab	6.1	CB	Normal	Normal	Normal	LOW	NK	LOW	NK	6	YES	Functional thyroid tests modified non-specifically. Clinical Euthyroidism. M1ADR	NO
5	C.E.	F	70	ADK	3	0	NEG	NEG	10%	T/LYM/M1OSS	Nivolumab	8.4	SD+CB	Normal	Normal	HIGH	LOW	NK	Normal	NK	3	NO	Basedow disease treated with Thyrozol. Current hypothyroidism	Levothyroxine
6	C.F.	B	67	ADK	1	0	NEG	NEG	90%	T/LYM	Pembrolizumab	14.7	PR+CB	Normal	Normal	Normal	Normal	NK	Normal	Normal	1	YES	Hypocorticism. Primary Adrenal Insufficiency due to steroids	NO
7	F.S.	B	65	ADK	1	0	NEG	NEG	NEG	NO	Pembrolizumab	9.1	PR	Normal	Normal	Normal	Normal	NK	LOW	NK	1	YES	Hypocorticism. Primary Adrenal Insufficiency due to steroids. Clinical Euthyroidism	NO
8	G.P.	B	78	SC	1	0	NK	NK	NEG	T/LYM	Pembrolizumab	11.2	PR+SD	Normal	Normal	LOW	HIGH	NK	NK	NK	4	YES	Hyperthyroxinemia related to iodinated contrast agent. Clinical Euthyroidism	NO
9	H.A.	B	65	SC	1	0	NEG	NK	NK	T/LYM/M1OSS	Atezolizumab	4.9	SD	Normal	Normal	HIGH	LOW	NK	NK	NK	3	NO	Therapeutically neglected hypothyroidism	Levothyroxine
10	I.P.	B	58	SC	2	0	NEG	NEG	8%	NO	Pembrolizumab	4.9	PR+SD	Normal	Normal	LOW	Normal	NK	NK	NK	1	NA	Hypercalcemia due to M1OSS	NO
11	P.C.	B	66	SC	3	0	NEG	NEG	50%	M1BRA	Pembrolizumab	6.3	PR	Normal	Normal	Normal	Normal	HIGH	NK	NK	1	NA	Clinical Euthyroidism. Non-thyrotoxic exophthalmia	NO
12	S.I.	B	66	ADK	1	0	NK	NEG	POZ	NO	Pembrolizumab	37.1	PR+SD	LOW	Normal	LOW	Normal	NK	NK	NK	2	YES	Central Hypothyroidism related to iodinated contrast agent. Minimal hypergonadotropic hypogonadism without indication of treatment	Levothyroxine
13	T.P.	B	67	SC	2	1	NEG	NEG	<1%	M1OSS	Pembrolizumab	5.6	PD	Normal	LOW	Normal	Normal	NK	NK	NK	3	NA	Micropolynodular Thyroid. Suspicion of M1 Other (parathyroid node). Clinical Euthyroidism.	NO
14	V.S.	F	65	SC	1	0	NEG	NEG	30%	M1BRA	Pembrolizumab	4.2	PR	Normal	Normal	LOW	LOW	NK	NK	NK	1	YES	Secondary Adrenal Insufficiency not-confirmed. Functional thyroid tests modified. Central Hypothyroidism. Clinical Euthyroidism.	NO

PFS, Progression-free survival; PR, Partial response; SD, Stable disease; CB, Clinical benefit, PD, Progressive disease, NA, Not applicable, NK, Not known, T, Tumor, LYM, Mediastinal lymph nodes, M1BRA, Brain metastasis, M1ADR, Adrenal metastasis, M1OSS, Bone metastasis; ICI, Immune check-point inhibitor. TPO Ab, thyroid peroxidase antibodies; ACTH, adrenocorticotropin; TSH, thyroid stimulating hormone; FT4, free thyroxine.

**Table 9:** Management of Hypophysitis and PAI according to Endocrinologist decision in our study.

Hypophysitis	Primary Adrenal Insufficiency
Grade 1–2: Mild to moderate symptoms	Grade 1–2: Mild to moderate symptoms
1. Hold ICI	1. Hold ICI
2. Initiate Hydrocortisone (15 mg/day) in 2 doses	2. Initiate Hydrocortisone (15 mg/day) in 2 doses
3. Initiate Levothyroxine 25 µg/day if central hypothyroidism also present	3. Monitor cortisol, ACTH (if possible) levels
4. Monitor TSH, FT4, cortisol, ACTH levels. If necessary monitor FSH, LH, testosterone.	4. Periodic endocrine reevaluation
5. Periodic endocrine reevaluation	5. Resume ICI when Hydrocortisone dose <10 mg/day
6. Resume ICI when Hydrocortisone dose <10 mg/day.	

**Table 10:** Management of Thyroid dysfunction according to Endocrinologist decision in our study.

Hypothyroidism	Hyperthyroidism
Grade 1: Asymptomatic or mild symptoms	Grade 1: Asymptomatic or mild symptoms
1. Continue ICI	1. Continue ICI
2. Initiate Levothyroxine 25 µg/day	2. Initiate symptomatic treatment if necessary
3. Monitor TSH, FT4 levels	3. Monitor TSH, FT4 levels
4. Periodic endocrine reevaluation	4. Periodic endocrine reevaluation

**Table 11:** Simple binary logistic regression.

Predictor	N (109)	irAEs N (13)	OR (95% CI) ^1^	*p*-Value
Sex				
M	76	9	—	
F	33	4	1.03 (0.26 to 3.43)	0.967
Age	109	13	0.96 (0.89 to 1.03)	0.236
Age over 65				
No	51	7	—	
Yes	58	6	0.73 (0.22 to 2.34)	0.588
HP				
ADK	76	10	—	
SC	33	3	0.66 (0.14 to 2.34)	0.549
No. of Meta	109	13	0.98 (0.53 to 1.69)	0.946
CHT				
No	22	3	—	
Yes	87	10	0.82 (0.23 to 2.59)	0.782
PDL1 > 50%				
No	58	6	—	
Yes	28	3	0.83 (0.24 to 2.54)	0.757
TSH during Treatment				
HIGH	8	6	—	
LOW	14	1	0.03 (0.01 to 0.25)	0.006
VN	84	6	0.03 (0.01 to 0.14)	<0.001
ICI Duration	109	13	1.02 (1.01 to 1.03)	0.008
No. of ICI Series	109	13	1.04 (1.01 to 1.07)	0.009

^1^ OR = odds ratio; CI = confidence interval.

**Table 12:** Multiple binary logistic regression.

Predictor	N (109)	NO-irAEs N (76)	OR (95% CI) ^1^	*p*-Value
TSH during Treatment				
HIGH	8	5	—	
LOW	14	11	3.43 (0.32 to 42.2)	0.31
Normal Values	84	60	1.20 (0.17 to 6.44)	0.84
ICI Duration (Weeks)	106	76	0.93 (0.88 to 0.97)	0.002
No. of ICI Series	106	76	1.14 (1.02 to 1.28)	0.022

^1^ OR = odds ratio; CI = confidence interval.

**Table 13:** Predictors of death.

Predictor	*N* (109)	Death *N* (79)	HR (95% CI) ^1^	*p*-Value
Sex				
M	76	54	—	
F	33	25	1.38 (0.85 to 2.24)	0.196
Age	109	79	1.03 (1.01 to 1.06)	0.037
Age over 65				
No	51	33	—	
Yes	58	46	1.40 (0.90 to 2.20)	0.139
HP				
ADK	76	55	—	
SC	33	24	1.29 (0.79 to 2.08)	0.306
PDL1 > 50%				
No	76	55	—	
Yes	33	24	1.29 (0.79 to 2.08)	0.306
No. of Meta	109	79	1.15 (0.94 to 1.41)	0.179
Surgery				
No	90	67	—	
Yes	19	12	0.48 (0.26 to 0.91)	0.023
CHT				
No	22	13	—	
Yes	87	66	1.66 (0.91 to 3.02)	0.096
irAEs				
No	96	72	—	
Yes	13	7	0.36 (0.16 to 0.80)	0.012

^1^ HR = hazard ratio; CI = confidence interval.

**Table 14:** Surgery and irAEs as predictors of death.

Predictor	N	Death N	HR (95% CI) ^1^	*p*-Value
Age	109	79	1.03 (1.00 to 1.06)	0.10
Surgery				
No	90	67	—	
Yes	19	12	0.49 (0.26 to 0.93)	0.032
irAEs				
No	96	72	—	
Yes	13	7	0.42 (0.19 to 0.93)	0.028

^1^ HR = hazard ratio; CI = confidence interval.

## Data Availability

The raw data supporting the conclusions of this article will be made available by the authors without undue reservation.
